# Comparison of Cumulative Dissipated Energy Between Two Phacoemulsification Systems Using a Modified Flip-and-Chop Technique With Multidirectional Jerking Movement From a Phacoemulsification Handpiece

**DOI:** 10.7759/cureus.118645

**Published:** 2026-10-26

**Authors:** Ming Chen, Hung-Yuan Lin, Emily J Kang

**Affiliations:** 1 Ophthalmology, John A. Burns School of Medicine, University of Hawaii, Honolulu, USA; 2 Ophthalmology, Zhongli University Eye Center, Taoyuan, TWN; 3 Medicine, John A. Burns School of Medicine, University of Hawaii, Honolulu, USA

**Keywords:** 4d phacoemulsification, cataract surgery, centurion, cumulative dissipated energy, flip and chop, phacoemulsification cataract surgery, unity

## Abstract

Introduction

The four-dimensional Unity phacoemulsification tip is designed to optimize tip motion and reduce time and energy consumption in cataract surgery. This study highlights a modified flip-and-chop technique with an added multidirectional jerking motion to the phacoemulsification handpiece intraocularly (referred to as the "five-dimensional flip-and-chop") and compares the cumulative dissipated energy (CDE) between the four-dimensional Unity system (Alcon Inc., Geneva, Switzerland) and the torsional Centurion system (Alcon Inc., Geneva, Switzerland) under this technique.

Methods

This is a retrospective, sequential, non-randomized comparison of 40 consecutive cases of Centurion to 40 consecutive cases of Unity performed by a single surgeon at a single ambulatory surgical suite. The initial cataract density between the groups was compared using optical coherence tomography (OCT) image quality (IQ) values from a single Maestro 2 device (Topcon, Tokyo, Japan).

Results

The two groups had comparable mean ages, sex distributions, and IQs. The mean CDE was significantly lower in the Unity group (5.39; SD=2.8) than in the Centurion group (8.23; SD=5.9), with p=0.0075.

Conclusion

Utilizing the modified "five-dimensional flip-and-chop" technique, the four-dimensional Unity system produced significantly lower CDE than the torsional Centurion system. The addition of this technique to the phacoemulsification handpiece may further improve the efficiency of phacoemulsification.

## Introduction

Phacoemulsification (phaco) was first developed by Charles Kelman in 1967. Since then, the cataractous lens can be emulsified through a small 2-3 mm incision [1,2]. There have been many modifications to the phaco system to improve the efficiency and safety of phaco cataract surgery. This study focuses specifically on the Centurion and Unity systems (Alcon Inc., Geneva, Switzerland).

The initial developments date back to 1970, when the tip of the phaco was one-dimensional and longitudinal only. In 2006, it was realized that the torsional movement of the tip can enhance the breakdown of the hard nucleus, and the two-dimensional phaco tip, such as the Centurion, was developed [3-9]. It was not until 2024 that a four-dimensional phaco tip with the Unity system was available to surgeons, achieving the goal of efficient phaco by optimizing tip motion, enhancing followability, reducing time, and reducing energy consumption in the eye and at the wound. A four-dimensional phaco tip provides multidirectional, repeatable pattern movement to achieve the goal. According to Alcon's manufacturer report, the four-dimensional phaco tip gives a 48% reduction in cumulative dissipated energy (CDE) and a 41% reduction in total energy consumption, with approximately two times faster nucleus removal [10]. The key difference between the two systems is that the Unity phaco tip moves in multiple directions during phaco, whereas the Centurion tip moves in only two.

CDE is a metric used in phaco to quantify the total ultrasound energy delivered to the eye during cataract surgery. It is measured in joules and reflects the sum of all energy dissipated by the phaco tip as it emulsifies the cataract nucleus. Bench and ex vivo models have been widely used to evaluate CDE and thermal dynamics under controlled conditions [8-10]. Lower CDE is associated with less intraocular trauma by phaco, potentially leading to better postoperative outcomes.

Classical techniques for phaco include divide-and-conquer, stop-and-chop, and phaco chop [1,2]. In 1991, Gimbel introduced divide-and-conquer, which involved dividing the nucleus into four quadrants by sculpting. Because of this method's safety and efficacy, it has become the standard technique taught to novice surgeons [1]. Nagahara, who followed in 1993, developed a method of manually fragmenting the nucleus without sculpting. This resulted in a significant reduction in phaco energy required [1,2]. Koch in 1994 first described dividing the nucleus with a groove, followed by subsequent chops of each heminucleus into smaller fragments. It did not yield the same results as the chopping technique [2]. In the last decade, the advent of the prechopped and femtosecond lasers has added to the options for nuclear management during cataract surgery in recent years [1,2]. The flip-and-chop technique, published in 2008 by Howard Fine, is based on the nucleus rotating freely within the capsular bag. A second instrument, introduced through the side-port incision, engages the subincisional nuclear rim and moves the inferior nuclear rim toward the center of the capsular bag for phaco [1,2].

In this paper, analogous to the four-dimensional phaco tip movement, a phaco handpiece controlled by the surgeon performs fine multidirectional jerking movements to efficiently remove the cataract. This jerk conceptually adds the fifth dimension, hereafter coining the term "five-dimensional flip-and-chop" technique. The Unity system's multidirectional phaco tip movement reduces surgical time and CDE compared with the two-directional Centurion system [10], and adding the surgeon's controlled jerking movements of the phaco handpiece may further reduce surgical time and CDE. The objective of this retrospective, sequential, non-randomized case series study is to disseminate this technique with a surgical video and compare the CDE between the two systems under the same modified technique.

## Materials and methods

All phaco cases were performed by a single surgeon using a flip-and-chop technique combined with a jerking movement by the same handpieces at Surgical Suite Hawaii with the same Centurion and Unity phaco systems (Video 1). 

**Video 1:** Modified flip-and-chop technique with multidirectional jerking movement of the phacoemulsification handpiece for five-dimensional phacoemulsification

This technique suggests performing a 5.5-6 mm capsulorhexis. The flip-and-chop technique is done by using cohesive viscoelastic to fill the space between the anterior capsule and nucleus for 360 degrees and balanced salt solution (BSS) to inject between the anterior capsule and nucleus for hydrodissection and to flip the nucleus out of the capsule bag. The volume of BSS for injection into hydrodissection needs to be carefully calculated, as too much solution may break the posterior capsule.

The sandwich technique is done by placing the dispersive viscoelastic between the nucleus and the corneal endothelium and between the nucleus and the posterior capsule. This technique protects the posterior capsule and corneal endothelium with the dispersive viscoelastic filling the spaces in front of and behind the nucleus after hydrodissection. The phaco tip should be bevel-down, with fine, deliberate, jerky movements centrally away from the endothelium and posterior capsule when performing phaco in the central anterior chamber. Gradual adaptation of these techniques, along with careful practice by surgeons experienced in flip-and-chop, is needed to ensure safety.

The settings were as follows: linear surgeon's control of aspiration (30-50 cc/min), linear control of power (15-50%), linear surgeon's control of vacuum (425-500 mmHg), inflow for pressure (50 cc/min), and the incision size of 2.4 mm.

While the standard unit of measurement of CDE is in joules, Centurion and Unity report in units of percent-seconds (%-sec), calculated as mean phaco power×phaco time. The CDE was calculated automatically by each phaco system (Alcon algorithm) and was recorded for each measurement. The CDE is a comparable measurement between the Unity and Centurion, two Alcon systems, because Alcon directly compared them in its data report [10].

The CDE of a group of 40 consecutive cases of phaco using the Centurion system in the first quarter of 2025 and another group of CDE of 40 consecutive cases of phaco using the Unity system in the first quarter of 2026 were retrieved from a computerized record at Surgical Suite Hawaii. The two groups are of the same mean age and sex.

All cases had preoperative optical coherence tomography (OCT) done by a single Maestro 2 device (Topcon, Tokyo, Japan). The image quality (IQ) data were extracted for all cases from the OCT report. The Lens Opacities Classification System III (LOCS III) was not used for the cataract density measurement due to the grading system not being a quantitative measurement and being subjective. Therefore, the IQ data from the Maestro 2 OCT were used as a reference for the quantitative comparison. The IQ is a numeric value for each scan to indicate how strong and reliable the signal is. It reflects the strength of the signal reaching the retina. A reduced number means the light has been scattered and absorbed by opacities, such as cataracts, along the path. The other parts of the media, like the cornea and vitreous, were clear in all eyes in the study. Images with poor signal, such as those with small pupils or patient movement, were excluded prior to the test. Therefore, the IQ value mainly reflects the lens opacity. Thus, the numeric values of the IQ were used as an objective, numeric measurement to estimate the density of cataract in this study.

In order to demonstrate that the cataract density of the two groups was the same, the mean IQ data of the two groups were compared using an unpaired t-test. The mean CDE of the Centurion group was compared to the mean CDE of the Unity group using an unpaired t-test. Statistical significance was set at p<0.05.

## Results

The mean age for each group was the same at 69.03 years (p=0.95), with an equal male-to-female ratio (Table 1).

**Table 1:** Centurion group vs. Unity group on mean age, OCT IQ, and CDE OCT: optical coherence tomography; IQ: image quality; CDE: cumulative dissipated energy

	Centurion group (n=40)	Unity group (n=40)	P-value
Mean age, years	69.03	69.03	0.95
OCT IQ, mean±SD	37.48±11.78	37.73±10.33	0.76
CDE, mean±SD	8.23±5.9	5.39±2.8	0.0075

The mean IQ for the Centurion group was 37.48 (SD=11.78) and for the Unity group 37.73 (SD=10.33) (p=0.76), indicating no statistically significant difference between the two groups for cataract density (Table 1, Figure 1).

**Figure 1:**
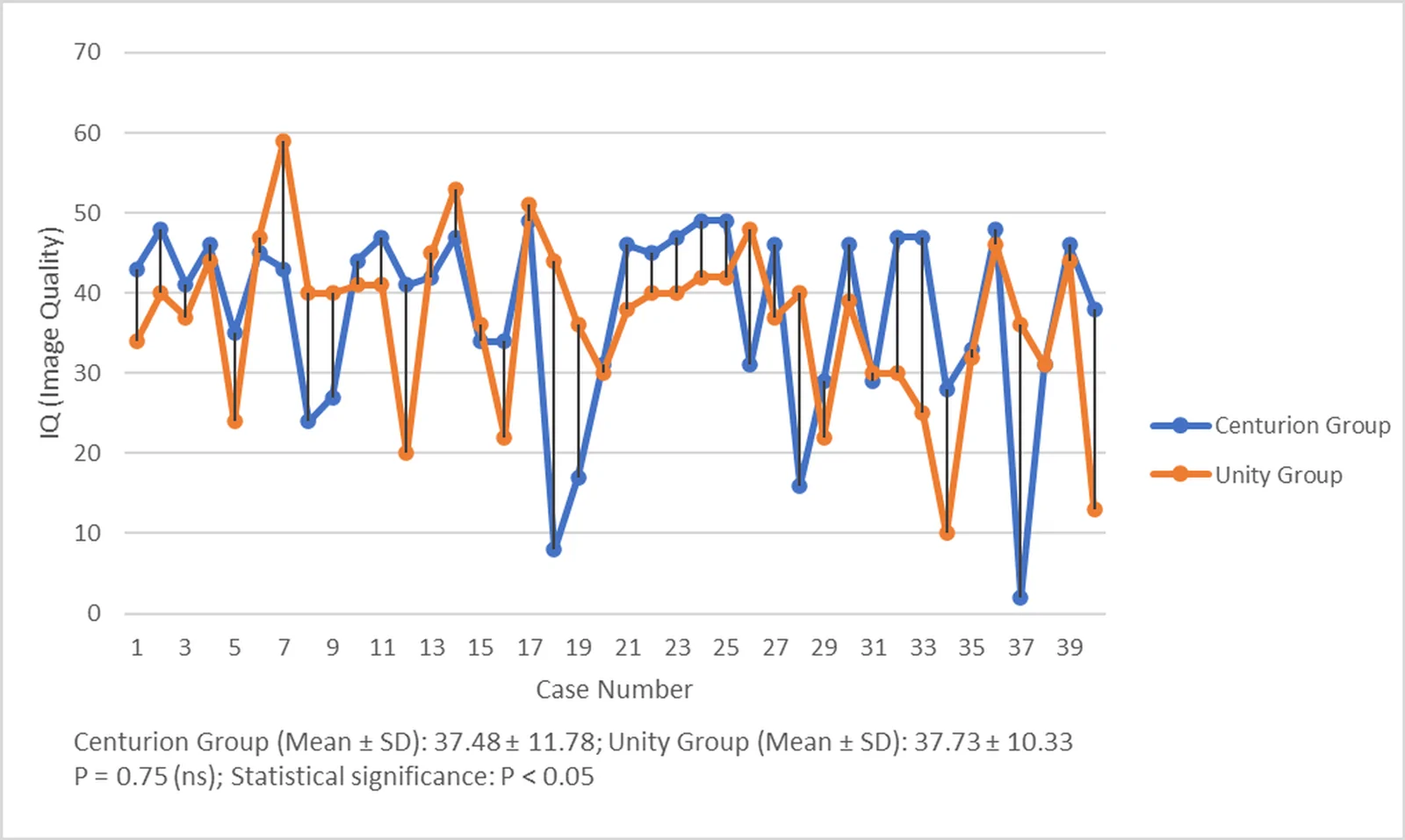
Centurion group vs. Unity group on OCT IQ for measuring cataract density The mean IQ data of the two groups (n=40 each) were compared using an unpaired t-test (p=0.76). OCT: optical coherence tomography; IQ: image quality

The mean CDE measured in percent-seconds was statistically lower in the Unity group with 5.39 (SD=2.8) than in the Centurion group with 8.23 (SD=5.9), with a 95% confidence interval of 0.78-4.89 and a p-value of 0.0075 (Table 1, Figure 2). The Centurion group included three high outliers (29.51, 30.7, 32.41), and the Unity group contained only one outlier (12.76).

**Figure 2:**
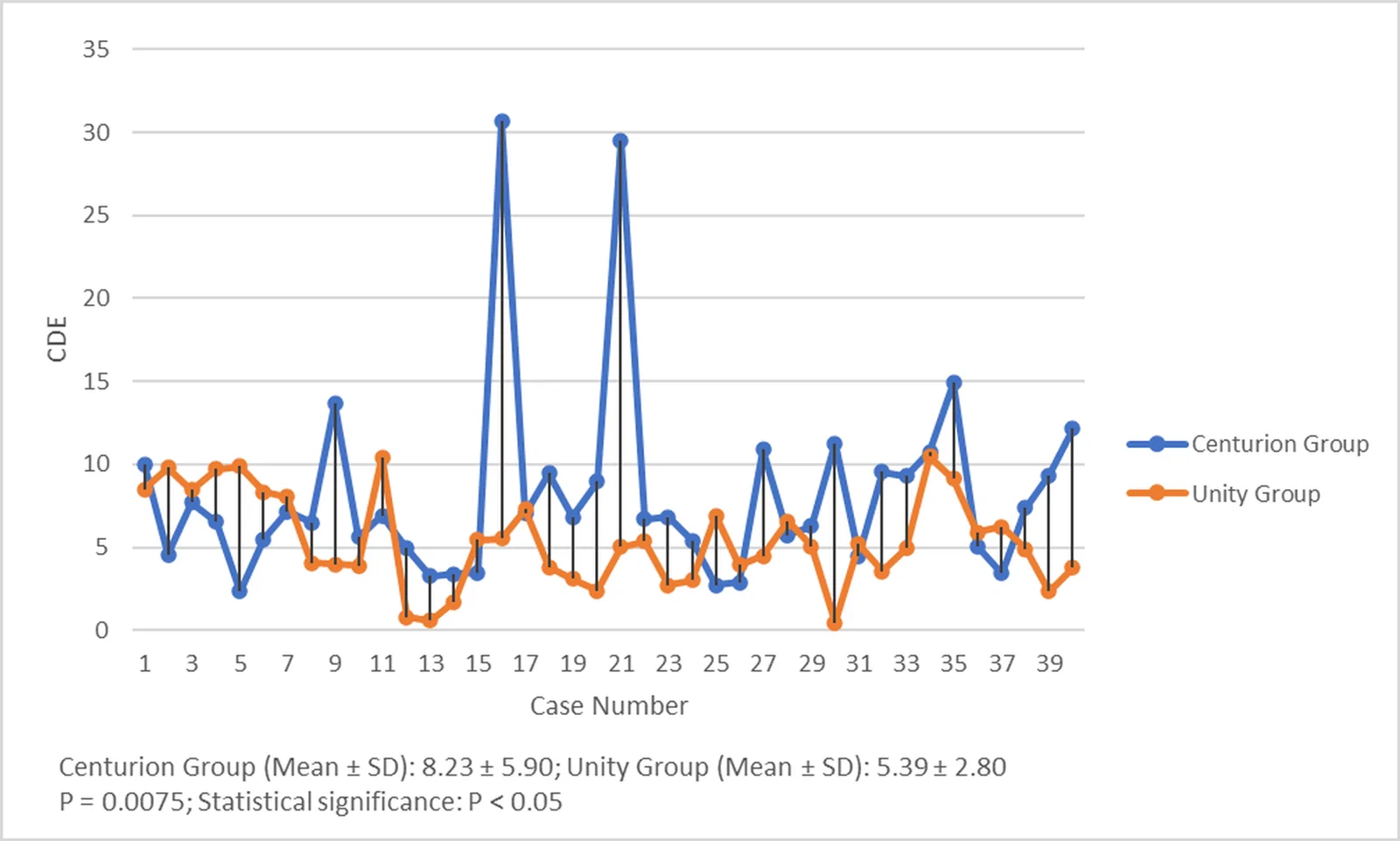
Centurion group vs. Unity group on CDE The mean CDE was significantly lower in the Unity group (n=40) than in the Centurion group (n=40) using an unpaired t-test (p=0.0075). CDE: cumulative dissipated energy

There were no surgical complications in either group. Second postoperative day examinations showed that most eyes had clear corneas, with a few eyes having corneal edema due to severe, dense cataracts.

## Discussion

Manufacturer data (Alcon) and other case reports indicate up to a 48% reduction in CDE and a 41% reduction in total energy consumption with Unity, as well as faster nucleus removal [10]. As Unity was available only for about one year for surgeons, there are few published papers available. A single case reported by Giglio and Tognetto of Italy showed the Unity CDE was 1.12 compared to Centurion of 15.01 on four divided dense cataract fragments of one patient on April 16, 2026 [11]. The clinical trial comparing two phaco systems in bilateral cataract surgery, sponsored by Nicole Fram of Los Angeles, recruiting 60 eyes of 30 subjects, is estimated to be completed on February 13, 2027. This study (NCT07468877) compares the Unity VCS/CS with four-dimensional phaco and the Centurion system with Ozil during bilateral cataract surgery, focusing on CDE and ultrasound time during cataract removal.

A separate prospective, randomized, paired-eye interventional study (NCT07222150) is designed to evaluate the effects of different intraocular pressure (IOP) settings during phaco cataract surgery on central corneal thickness (CCT) and corneal clarity at day 1 and day 8 post-operation by comparing Unity (IOP <28) and Centurion (IOP >65).

This study included 80 cases in similar groups, as evidenced by the same mean age and sex distribution, and all patients belonged to the same clinic during a similar sequential time period. The comparison of mean CDE between Unity and Centurion by a single surgeon, using a novel single technique at a single center, demonstrated the statistical significance of the lower CDE with Unity's phaco tip. The study also showed that this modified flip-and-chop technique, with the jerking movement of the phaco handpiece controlled by the surgeon, may further reduce the CDE. Some experienced cataract surgeons may already use this jerking technique; nevertheless, it needs to be disseminated to younger cataract surgeons to enhance their technique.

There are limitations to this study as it is a retrospective case series data analysis. To focus on the retrospective comparison of CDE between the two systems, other eye examination data, such as vision, IOP, endothelial cell count, corneal clarity, and retinal findings, were not included in this paper. Also, the sequential sampling of the study design may impose confounding factors such as increased surgeon experience over time. Nevertheless, the similarity of the two groups was demonstrated for meaningful comparison. The preoperative cataract density measurement was not done by the LOCS III system through slit lamp. However, the IQ data from the Maestro 2 OCT was used for the comparison of cataract density since all subjects had pre-op OCT with clear cornea and vitreous. It is important to note as a limitation that the OCT IQ may be reduced in eyes with dense posterior subcapsular cataract and this may affect the precision of density estimation in such dense, severe cataract cases. The age, sex, and population in comparable periods in two consecutive years, with all patients being the same surgeon's patients and the mean IQ data between the two groups not being statistically different, support the conclusion that the cataract density was similar. Furthermore, without the direct comparison of a control group in this study without the jerking movement, the CDE reduction between Unity and Centurion cannot be attributed to the addition of the jerking movement or to the effect of the difference in system. Lastly, this study does not include postoperative outcome data such as visual acuity and endothelial cell count, which, if available, would directly show the clinical benefits of lower CDE.

The noteworthy point in this paper is the combination of the novel surgeon-controlled phaco handpiece jerking technique combined with the four-dimensional phaco tip, which conceptually adds a fifth dimension of movement. CDE was significantly lower with the Unity system when used with this technique, though the independent contribution of the jerking movement requires further study. Furthermore, this paper introduces the use of IQ data from Maestro 2 OCT as a novel way of measuring the density of cataract if other objective density measurement equipment such as densitometry or Scheimpflug cameras are not available.

No surgical complications were reported in either group, suggesting that the novel flip-and-chop technique may be safe when performed by an experienced surgeon.

A future comparison of CDE across different phaco techniques, including the modified flip-and-chop technique using the Unity system, to elucidate which technique is best at reducing CDE is warranted. Furthermore, a future study of a four-group comparison of Centurion and Unity, each with and without the jerking movement, would be an ideal design to isolate and further characterize the effect of the added jerking movement technique from that of the platform.

## Conclusions

The four-dimensional tip of Unity on the phaco tip can significantly reduce mean CDE compared to the two-dimensional torsional tip of Centurion. The addition of the modified flip-and-chop with jerking movement on the phaco handpiece may further improve the efficiency of phaco.
